# Efficient activation of T cells by human monocyte-derived dendritic cells (HMDCs) pulsed with *Coxiella burnetii *outer membrane protein Com1 but not by HspB-pulsed HMDCs

**DOI:** 10.1186/1471-2172-12-52

**Published:** 2011-09-03

**Authors:** Ying Wang, Xiaolu Xiong, Deping Wu, Xile Wang, Bohai Wen

**Affiliations:** 1 State Key Laboratory of Pathogen and Biosecurity, Beijing Institute of Microbiology and Epidemiology, Beijing 100071, China; 2 The 457th hospital of PLA, Wuhan 430012, China; 3 The 82nd hospital of PLA, Huaian 223001, China

## Abstract

**Background:**

*Coxiella burnetii *is an obligate intracellular bacterium and the etiologic agent of Q fever; both coxiella outer membrane protein 1 (Com1) and heat shock protein B (HspB) are its major immunodominant antigens. It is not clear whether Com1 and HspB have the ability to mount immune responses against *C. burnetii *infection.

**Results:**

The recombinant proteins Com1 and HspB were applied to pulse human monocyte-derived dendritic cells (HMDCs), and the pulsed HMDCs were used to stimulate isogenic T cells. Com1-pulsed HMDCs expressed substantially higher levels of surface molecules (CD83, CD40, CD80, CD86, CD54, and CD58) and a higher level of interleukin-12 than HspB-pulsed HMDCs. Moreover, Com1-pulsed HMDCs induced high-level proliferation and activation of CD4^+ ^and CD8^+ ^cells, which expressed high levels of T-cell activation marker CD69 and inflammatory cytokines IFN-γ and TNF-α. In contrast, HspB-pulsed HMDCs were unable to induce efficient T-cell proliferation and activation.

**Conclusions:**

Our results demonstrate that Com1-pulsed HMDCs are able to induce efficient T-cell proliferation and drive T cells toward Th1 and Tc1 polarization; however, HspB-pulsed HMDCs are unable to do so. Unlike HspB, Com1 is a protective antigen, which was demonstrated by the adoptive transfer of Com1-pulsed bone marrow dendritic cells into naive BALB/c mice.

## Background

Dendritic cells (DCs) are potent antigen-presenting cells (APCs) that bridge the innate and adaptive immune responses through direct pathogen neutralization, cytokine production, and T-cell activation [1]. Activated DCs express major histocompatibility complex (MHC) I and MHC II molecules and T-cell costimulatory molecules, which possess the unique ability to activate naive T cells [2,3]. Immature DCs (iDCs) reside in the peripheral epithelial tissues, where they serve as sentinels against invading microorganisms [4]. Contact with a pathogen typically elicits stimulation of iDCs via pattern-recognition receptors, such as the toll-like receptor (TLR), and subsequent conversion of iDCs to mature DCs (mDCs) [5,6]. mDCs exhibit a reduction in phagocytic ability and an increase in surface expression of MHC II and costimulatory molecules, and they switch in chemokine receptor expression, which results in mDC migration to the local lymph nodes to induce adaptive immunity [2,7,8].

*Coxiella burnetii *is a Gram-negative, obligate intracellular bacterium, which survives inside large replication vacuoles that display phagolysosomal characteristics [9,10]. *C. burnetii *is the etiologic agent of Q fever, a disease with a worldwide distribution [11,12]. Acute Q fever is usually self-limited in immunocompetent hosts, whereas the chronic form of the disease develops in individuals defective in cell-mediated immunity [11,13]. Owing to its very low infectious dose, known environment stability, and aerosol transmission route, *C. burnetii *is recognized as a potential biological weapon agent, and it has been classified as a category B bioterrorism agent [13,14]. An inactivated *C. burnetii *vaccine against acute Q fever is effective, but the vaccination remains problematic owing to its significant side effects in individuals who have already had Q fever [15]. Second-generation Q fever vaccine, offering significant relief from adverse reactions, has been developed by means of chloroform-methanol extraction of phase I organisms by Williams and colleagues [16,17]; however, the complex procedure and biosecurity requirements in dealing with the propagation and purification of *C. burnetii *organisms are a hindrance to large-scale production.

A safe, effective subunit vaccine against Q fever would clearly be desirable, and a number of potential protective antigens have been identified toward developing such a vaccine [18-22]. Among them, coxiella outer-membrane protein 1 (Com1) and heat-shock protein B (HspB) were strongly recognized by sera from Q fever patients or *C. burnetii-*infected animals [20-22]. HspB is a member of the Hsp60 family, and Hsp60 in *Legionella pneumophila *(a facultative intracellular pathogen largely similar to *C. burnetii *in phylogenesis) is located in the periplasm and outer membrane, and the surface-associated Hsp60 is involved in bacterial virulence [23,24]. Both Com1 and HspB of *C. burnetii *are viewed as major immunodominant antigens and important surface-associated molecules that can interact with surface molecules of innate/adaptive immune cells [18-20,25]. However, it is not clear whether Com1 and HspB possess the ability to mount immune responses against *C. burnetii *infection.

In the present study, the recombinant proteins Com1 and HspB were used to pulse human monocyte-derived DCs (HMDCs) in vitro, and the maturation and activation status of the pulsed HMDCs were measured by flow cytometry. Since the protective response to intracellular bacteria is mainly dependent on a cell-mediated immune reaction, the pulsed HMDCs were used to stimulate isogenic T cells in vitro; the maturation and activation status of CD4^+ ^and CD8^+ ^cells in the T-cell population were analyzed after interaction of the HMDCs with the T cells. We found that Com1 induced efficient maturation and activation of HMDCs and that Com1-pulsed cells were able to initiate the adaptive T-cell response by skewing CD4^+ ^and CD8^+ ^cells toward, respectively, the Th1 and Tc1 pathway. In contrast, HspB elicited incomplete maturation of HMDCs, and HspB-pulsed cells were unable to induce efficient T-cell proliferation and activation.

## Results

### Immunoblot analysis of Com1 and HspB

**Figure 1:**
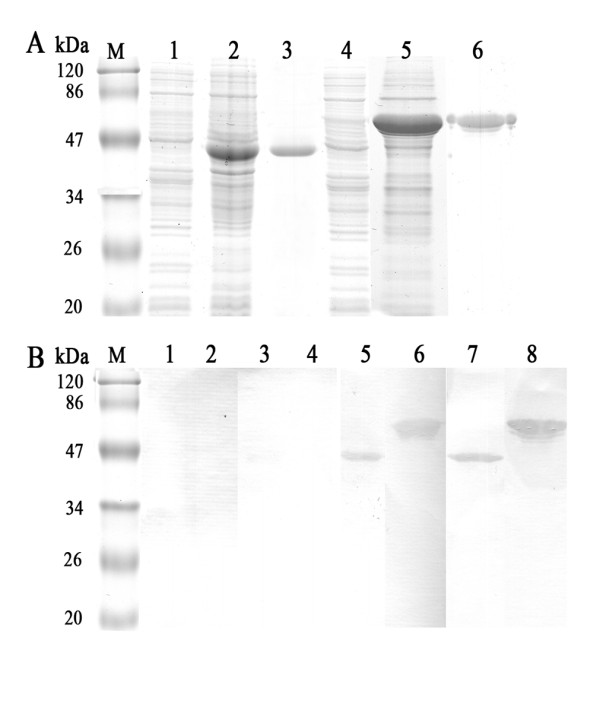
**SDS-PAGE and immunoblot analysis of recombinant proteins Com1 and HspB**. A SDS-PAGE analysis of Com1 and HspB: lane M, protein molecular mass markers; lane 1, lysate of *E. coli *cells harboring plasmid; lane 2, lysate of *E. coli *cells harboring *com1*-recombined plasmid; lane 3, purified 41 kDa Com1 fused with 17 kDa tagged-peptide from pET32a expression plasmid; lane 4, lysate of *E. coli *cells harboring plasmid; lane 5, lysate of *E. coli *cells harboring *hspB*-recombined plasmid; lane 6, purified 54 kDa HspB. B Immunoblot analysis of proteins Com1 and HspB with sera from mice experimentally infected with *C. burnetii*. Lanes 1, 3, 5, and 7, Com1 immunoblot assay with mouse sera obtained at days 7, 14, 21, and 28 postinfection, respectively, with *C. burnetii*. Lanes 2, 4, 6, and 8, HspB immunoblot assay with mouse sera obtained at days 7, 14, 21, and 28, respectively, postinfection with *C. burnetii*.

The purified recombinant proteins Com1 and HspB were analyzed by sodium dodecylsulfate polyacrylamide gel electrophoresis (SDS-PAGE; Figure 1A) and incubated with sera from mice experimentally infected with *C. burnetii *(Figure 1B). The recombinant Com1 and HspB were strongly recognized by infected mouse sera obtained at 21 and 28 days postinfection.

### Maturation and activation of HMDCs induced by Com1 and HspB

**Figure 2:**
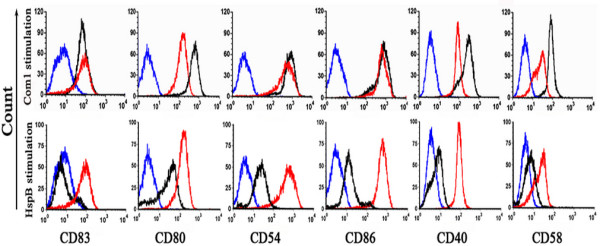
**Expression of surface molecules on antigen-treated HMDCs**. After 24 h of Com1 or HspB stimulation (black histograms), HMDCs were stained with monoclonal antibody to CD83, CD80, CD54, CD86, CD40, or CD58 and the expression of the surface molecules was measured by flow cytometry. LPS-treated HMDCs (red histograms) and mock-stimulated HMDCs (blue histograms) were applied as positive and negative controls, respectively. The results are representative of three independent experiments, each using cells derived from a different donor.

To determine the capability of Com1 and HspB to induce maturation and activation of HMDCs, immature HMDCs (iHMDCs) were treated with Com1, HspB, or *Escherichia coli *lipopolysaccharide (LPS) for 24 h. The resulting populations of HMDCs were analyzed by flow cytometry to determine their expression of surface molecules. As shown in Figure 2, Com1-treated HMDCs exhibited substantially increased expression of surface molecules, and the expression levels of surface molecules (CD40, CD54, CD58, CD80, CD83, and CD86) were equal to or higher than those of LPS-treated HMDCs. However, with HspB-treated HMDCs, CD83 expression remained at baseline levels, while expression of the other surface molecules was much lower than with Com1-pulsed HMDCs (Figure 2).

### Cytokine expression of HMDC elicited by Com1 and HspB

**Figure 3:**
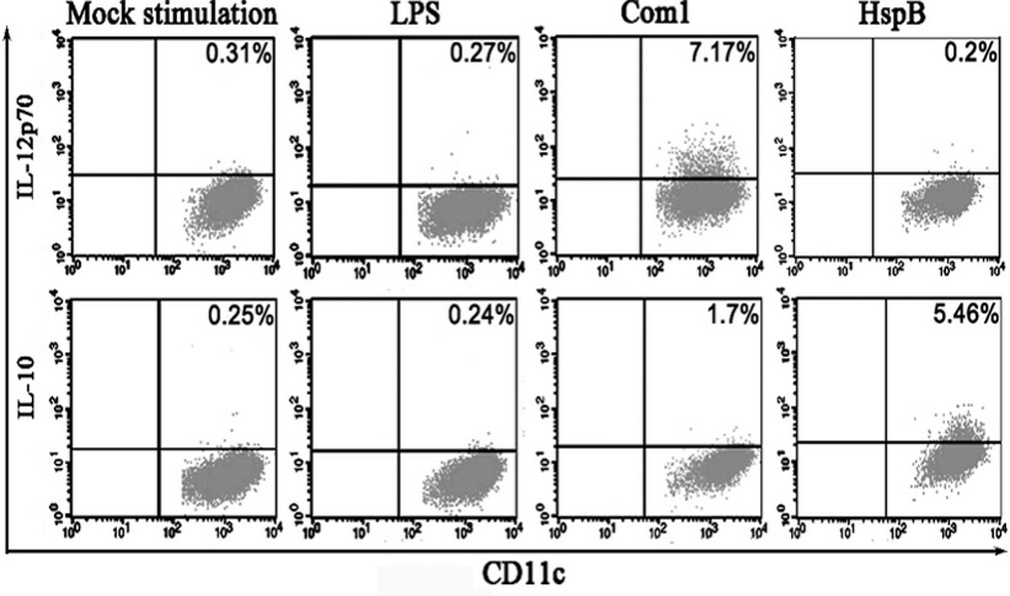
**Production of IL-12p70 and IL-10 in antigen-treated HMDCs**. After 24 h of stimulation with *C. burnetii *antigens, IL-12p70 and IL-10 expression in CD11c^+ ^HMDCs were analyzed by intracellular cytokine staining. The percentages of double-positive cells in HMDCs are indicated in the top-right corner, and the results are representative of three independent experiments, each using cells derived from a different donor.

Antigen-activated DCs play an important role in controlling differentiation orientation of naive T cells by differential expression of IL-12 and IL-10. To investigate the possible effector function of Com1- or HspB-pulsed HMDCs on T cells, the production of intracellular IL-12p70 and IL-10 was determined in the antigen-treated HMDCs. As seen in Figure 3, the percentages of IL-12 p70-positive cells in the Com1-pulsed HMDCs were approximately 23-, 30-, and 35-fold higher, respectively, than in the mock-pulsed HMDCs, LPS-pulsed HMDCs, and HspB-pulsed HMDCs. The percentage of IL-12p70-positive cells in the HspB-pulsed HMDCs was 36-fold lower than in the Com1-pulsed HMDCs, whereas the percentage of IL-10-positive cells in the HspB-pulsed HMDCs was threefold higher than in the Com1-pulsed HMDCs (Figure 3).

### Early T-cell activation induced by antigen-pulsed HMDCs

**Figure 4:**
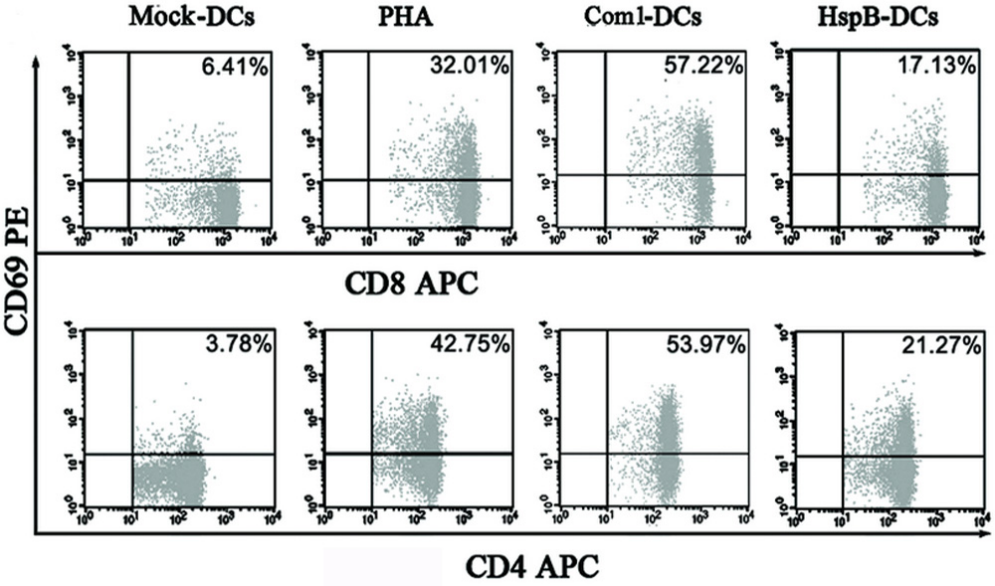
**CD69 expression in T cells interacted with antigen-pulsed HMDCs**. After coculture with antigen-pulsed HMDCs (Com1-DCs/HspB-DCs), mock-pulsed HMDCs (Mock-DCs), or PHA (positive control), CD69 expression on the surfaces of CD4+ and CD8+ cells were determined in T cells. The percentages of double-positive cells among the T cells are indicated in the top-right corner, and the results are representative of three independent experiments, each using cells derived from a different donor.

To evaluate the direct effect of the antigen-pulsed HMDCs on T-cell activation, CD69 expression in both CD4^+ ^and CD8^+ ^cells was determined by flow cytometry after 12 h of interaction of T cells with antigen-pulsed HMDCs (Figure 4). As a result, the percentages of CD4-CD69 and CD8-CD69 double-positive cells among T cells cocultured with Com1-pulsed HMDCs were 8.9- and 14.2-fold higher, respectively, than those cocultured with mock-pulsed HMDCs; the percentages of CD4-CD69 and CD8-CD69 double-positive cells among T cells cocultured with HspB-pulsed HMDCs were only 2.7- and 5.6-fold higher, respectively, than those cocultured with mock-pulsed HMDCs (Figure 4). The percentages of CD4-CD69 and CD8-CD69 double-positive cells among T-cells cocultured with Com1-pulsed HMDCs were 3.3- and 2.5-fold higher, respectively, than those cocultured with HspB-pulsed HMDCs (Figure 4).

### Cytokine production of T cells elicited by antigen-pulsed HMDCs

**Figure 5:**
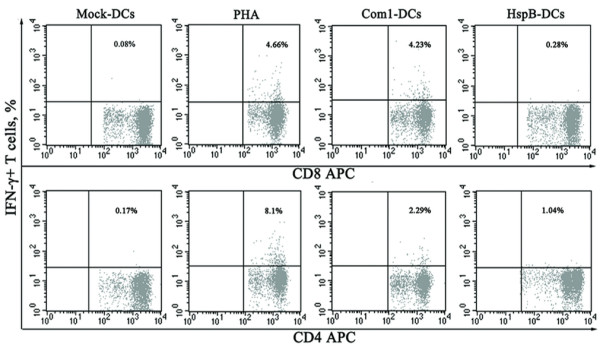
**IFN-γ expression in CD4^+ ^and CD8^+ ^cells induced with antigen-pulsed HMDCs**. IFN-γ expression in CD4^+ ^and CD8^+ ^cells was analyzed in T cells cocultured with antigen-pulsed HMDCs (Com1-DCs/HspB-DCs), mock-pulsed HMDCs (mock-DCs), or PHA (positive control) by intracellular IFN-γ staining. The percentages of double-positive cells among the T cells are indicated in the top-right corner, and the results are representative of three independent experiments, each using cells derived from a different donor.

**Figure 6:**
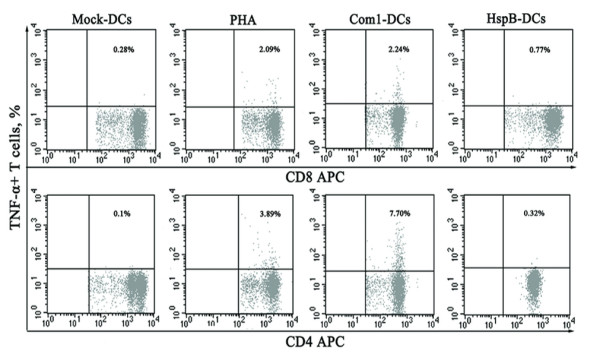
**TNF-α expression in CD4^+ ^and CD8^+ ^cells induced with antigen-pulsed HMDCs**. TNF-α expression on CD4^+ ^and CD8^+ ^cells was analyzed in T cells cocultured with antigen-pulsed HMDCs (Com1-DCs/HspB-DCs), mock-pulsed HMDCs (mock-DCs), or PHA (positive control) by intracellular TNF-α staining. The percentages of double-positive cells among the T cells are indicated in the top-right corner, and the results are representative of three independent experiments, each using cells derived from a different donor.

To determine the differentiation orientation of T cells after their interaction with antigen-pulsed HMDCs, the production of cytokines (IFN-γ, TNF-α, and IL-10) was determined in CD4^+ ^and CD8^+ ^cells. The percentages of CD4-IFN-γ and CD8-IFN-γ double-positive cells among T-cells cocultured with Com1-pulsed HMDCs were 2.2- and 15.1-fold higher, respectively, than those cocultured with HspB-pulsed HMDCs (Figure 5). Meanwhile, the percentages of CD4-TNF-α and CD8-TNF-α double-positive cells among T cells cocultured with Com1-pulsed HMDCs were 24.1- and 2.9-fold higher, respectively, than those cocultured with HspB-pulsed HMDCs (Figure 6). Additionally, IL-10 expression of CD4^+ ^and CD8^+ ^cells among T cells treated with Com1-pulsed HMDCs or HspB-pulsed HMDCs remained at baseline levels (data not shown).

### T-cell proliferation induced by antigen-pulsed HMDCs

**Figure 7:**
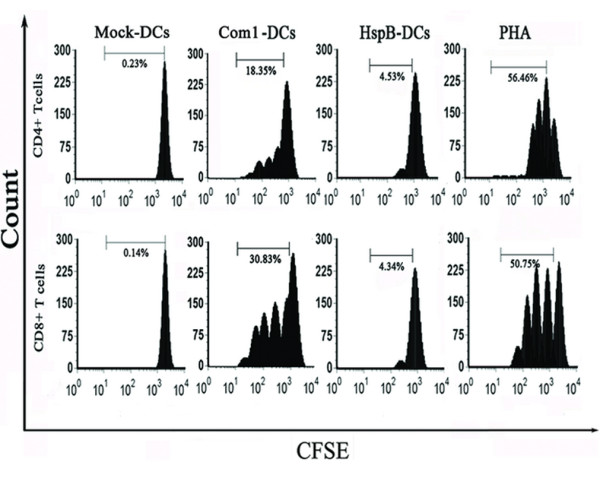
**Proliferation of CD4^+ ^and CD8^+ ^cells in response to antigen-treated HMDCs**. The CFSE-stained T cells were stimulated with antigen-treated HMDCs (Com1-DCs/HspB-DCs), mock-pulsed HMDCs (Mock-DCs), or PHA (positive control) for 96 h. The results are representative of three independent experiments, each using cells derived from a different donor.

T-cell proliferation induced by Com1- or HspB-pulsed HMDCs was evaluated by carboxyfluorescein diacetate succinimidyl ester (CFSE)-based proliferation assay after 96 h of interaction of T cells with antigen-pulsed HMDCs. The proliferation rates of CD4^+ ^and CD8^+ ^cells among T-cells cocultured with Com1-pulsed HMDCs were 4.1- and 7.1-fold higher, respectively, than those cocultured with HspB-pulsed HMDCs (Figure 7).

### Immune protective analysis of Com1- and HspB-pulsed BMDCs

**Figure 8:**
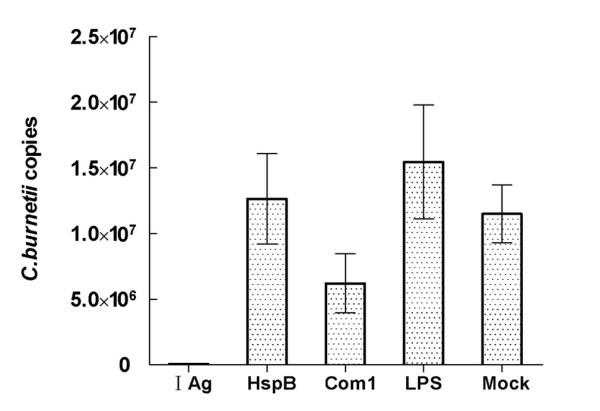
**Estimation of coxiella burden in recipient mice of antigen-pulsed BMDCs**. Coxiella DNA copies were determined in five mouse samples per group by quantitative PCR. Data are expressed as the average copy number of five samples, and error bars indicate the standard deviation. Mice receiving phase I Ag-pulsed BMDCs (I Ag) and mock-pulsed BMDCs (Mock) were used as positive and negative controls, respectively. In quantitative PCR analysis, the copy number per mouse was obtained with 1% of the DNA sample extracted from 10 mg spleen tissue. Coxiella burden in mice receiving Com1-pulsed BMDCs was significantly lower than in mice receiving HspB-pulsed BMDCs (*P*<0.05). This result is from one independent experiment.

Compared with mice receiving mock-pulsed BMDCs (control), animals receiving Com1-pulsed BMDCs exhibited significantly lower coxiella burden; mice receiving HspB-pulsed BMDCs displayed similar high levels of coxiella burden after challenge with *C. burnetii *(Figure 8). Coxiella burden in mice receiving HspB-pulsed BMDCs was significantly higher than in those receiving Com1-pulsed BMDCs; coxiella burden in animals receiving I Ag-pulsed BMDCs was much lower than in mice receiving Com1-pulsed BMDCs (Figure 8).

## Discussion

Com1 was first recognized as an immunoreactive protein associated with the outer membrane of *C. burnetii *by Hendrix and colleagues [19], and it was identified as a major immunodominant antigen of *C. burnetii *in other studies [20,21]. HspB was similarly recognized as a major immunodominant antigen of *C. burnetii *[20] and was suggested as being a surface-associated protein [24]. However, it is unclear whether Com1 and HspB are able to mount specific immune responses, particularly a cell-mediated immune response against *C. burnetii *in humans.

In the present study, Com1 and HspB were applied to stimulate HMDCs, and the antigen-pulsed HMDCs were arranged to stimulate isogenic T cells. To explore a possible mechanism of the immune response induced by Com1 or HspB, the immune effector functions (including T-cell activation-associated cytokines expressed in the antigen-pulsed HMDCs and the cytokines associated with intracellular bactericidal activities in the T cells activated by the antigen-pulsed HMDCs) were measured by flow cytometry.

DCs are present in most tissues in an immature status, and they undergo maturation upon antigen capture, whereupon they migrate to the lymph nodes to elicit adaptive immune responses [1]. In the present study, the Com1-pulsed HMDCs exhibited increased expression of surface molecules, including the DC-maturation marker (CD83), costimulatory molecules (CD40, CD80, and CD86), and adhesion molecules (CD54 and CD58). In addition, CD83, CD80, and CD86 are DC-maturation markers, and thus their increased expression in Com1-pulsed HMDCs suggests that Com1 has the ability to induce efficient maturation of HMDCs.

These surface molecules expressed on mature HMDCs may efficiently initiate the primary adaptive immune response. During this response, naive T cells polarize toward the antigen-presenting DCs, and a specific large-scale molecular complex is built at the DC-T-cell interface [26]. T-cell activation is regulated primarily by signalling events that derive from the interaction between the T-cell receptor (TCR) and the presented antigens on DCs and the interaction between T-cell surface molecules (CD28, CD154, CD2, and CD11a) and costimulatory (CD40, CD80, and CD86) or adhesion molecules (CD54 and CD58) on DCs [27]. The CD80/CD86-CD28 interaction provides important signals for T-cell activation and survival, and the CD40-CD154 interaction is crucial for the development of CD4^+ ^T-cell-dependent effector functions; the interactions of both CD54-CD11a and CD58-CD2 act as a central part in the clustering of DCs and CD4^+ ^T cells [27]. Therefore, the increased expression of costimulatory and adhesion molecules on Com1-activated HMDCs strongly indicates that Com1-activated HMDCs possess the ability to induce efficient proliferation and activation of naive T cells.

T-cell proliferation induced by antigen-treated HMDCs was measured by flow cytometry of CFSE dye dilution. Our results showed that the proliferation levels of CD4^+ ^and CD8^+ ^cells in T cells cocultured with Com1-activated HMDCs were significantly higher than those cocultured with mock-pulsed HMDCs. This suggests that Com1-activated HMDCs efficiently stimulated naive T cells to expand the populations of both CD4^+ ^and CD8^+ ^subsets before acquiring T-cell effector function.

It is well established that antigen-activated DCs can regulate the balance of Th1 and Th2 by differential expression between IL-12 and IL-10. If IL-12 is much higher than IL-10, the naive T cells will be skewed toward the Th1 phenotype; conversely, if IL-10 is much higher than IL-12, the naive T cells will differentiate toward the Th2 phenotype. In the present study, high IL-12p70 and low IL-10 expression were found in Com1-pulsed HMDCs, which suggests that Com1-activated HMDCs possess the ability to drive T cells toward Th1 differentiation through the Th1-specific cytokine IL-12.

To demonstrate the capability of Com1-pulsed HMDCs to activate T cells, the T-cell activation marker CD69 on CD4^+ ^and CD8^+ ^cells was determined after T-cell interaction with Com1-pulsed HMDCs. Our results showed that the percentages of CD4-CD69 and CD8-CD69 double-positive cells among T cells that interacted with Com1-pulsed HMDCs were much greater than those that interacted with mock-pulsed HMDCs. This firmly suggests that both CD4^+ ^and CD8^+ ^cells are efficiently activated through strong accessory signals derived from the surface-molecule interactions between Com1-activated HMDCs and T cells.

Laboratory studies have demonstrated that protection against *C. burnetii *infection is conferred by a Th1-oriented immune response, which depends on the Th1-specific cytokine IFN-γ and inflammatory cytokine TNF-α since both cytokines have the ability to upregulate the bactericidal activity of macrophages [28,29]. In the present study, Th1-specific cytokine IFN-γ and Th2-specific cytokine IL-10 as well as inflammatory cytokine TNF-α were determined in T cells after exposure to Com1-activated HMDCs. Our results showed that the expression of IFN-γ and TNF-α in CD4^+ ^and CD8^+ ^cells was dramatically increased after T-cell interaction with Com1-pulsed HMDCs. However, the IL-10 expression in CD4^+ ^and CD8^+ ^cells in this T-cell population remained at baseline levels. These results strongly imply that Com1-pulsed HMDCs have the ability to drive differentiation of naive CD4^+ ^and CD8^+ ^cells toward, respectively, CD4^+ ^Th1 and CD8^+ ^Tcl cells and that HMDC-activated CD4^+ ^and CD8^+ ^cells may produce IFN-γ and TNF-α to enhance the intracellular bactericidal activity of the host cells (macrophages).

To demonstrate the immunoprotective ability of Com1, Com1-pulsed BMDCs were adaptively transferred to naive mice. Our results showed that mice receiving Com1-pulsed BMDCs exhibited significantly lower coxiella burden than control animals after challenge with virulent *C. burnetii*. However, the coxiella burden in mice receiving HspB-pulsed BMDCs was significantly higher than in those receiving Com1-pulsed BMDCs. This suggests that HspB is not a protective antigen.

We found that HspB was unable to induce efficient maturation and activation of HMDCs. The expressions of maturation marker (CD83), costimulatory (CD40, CD80, and CD86), and adhesion molecules (CD54 and CD58) of HspB-pulsed HMDCs were much lower than those of Com1-pulsed HMDCs. Moreover, HspB-pulsed HMDCs produced much lower levels of IL-12, but much higher levels of IL-10, than Com1-pulsed HMDCs. The increased expression of IL-10 in DCs may inhibit T-cell proliferation [30,31]. Similarly, Motta et al. [32] showed that *Mycobacterium tuberculosis *Hsp70 inhibited T-cell proliferation in vitro, since it impaired DC maturation and induced high production of IL-10 in DCs; this suggests that Hsp70 does not have inflammatory potential but rather immunosuppressive properties.

In this study, the percentages of CD8-CD69 and CD4-CD69 double-positive cells among T cells cocultured with HspB-pulsed HMDCs were much lower than those cocultured with Com1-pulsed HMDCs. HspB-pulsed HMDCs caused a negligible proliferation of CD8^+ ^and CD4^+ ^cells, which suggests that the low-level expression of costimulatory molecules and adherence molecules of HspB-pulsed HMDCs was unable to activate the T cells.

Generally, the interaction between the innate/adaptive immune system and invading *C. burnetii *is sufficient to eradicate the organisms, which elicit asymptomatic or mild, self-limiting, flu-like symptoms in most human *C. burnetii *infections. The bactericidal immune response is mainly based on inflammatory cytokines IFN-γ and TNF-α [33,34]. However, a variety of pathogens can take advantage of the anti-inflammatory properties of IL-10 (which is crucial for maintaining the subtle balance between immunity against pathogens and a pathology that can arise when an immune response is left unchecked) as a mean of avoiding sterilizing immunity and establishing a persistent infection [35-37]. In *C. burnetii *infections, IL-10 is believed to have the ability to disarm bactericidal responses and contribute to chronic development of *C. burnetii *infection. Previous studies [38,39] showed that IL-10 promoted replication of *C. burnetii *in human monocytes and *C. burnetii *established a more robust infection in IL-10-overexpressing transgenic mice owing to the macrophages failing to kill the bacteria. Another study revealed that IL-10 overproduction was involved in establishing persistent *C. burnetii *infections and was related to Q fever endocarditis development [40]. Clearly, IL-10 plays an important role in persistent *C. burnetii *infection. Therefore, the high level of anti-inflammatory cytokine IL-10 generated in HspB-pulsed HMDCs may be an important factor in inhibiting proliferation and activation of T cells.

In our recent study, adaptive transfer of mouse BMDCs stimulated with coxiella antigens into BABL/c mice showed that the coxiella burden in animals receiving Com1-pulsed BMDCs was significantly lower than in mice receiving EnhA-pulsed BMDCs [41]. Although both Com1 and EnhA had the ability to activate BMDCs to express MHC-II and costimulatory molecules, CD4^+ ^T cells from mice receiving Com1-pulsed BMDCs exhibited significantly higher CD69 expression than animals receiving EnhA-pulsed BMDCs after coculture with homologous antigen-pulsed BMDCs. In addition, the percentages of CD4^+ ^and IFN-γ double-positive cells in mice receiving Com1-pulsed BMDCs were significantly higher than in animals receiving EnhA-pulsed BMDCs; the percentages of CD4^+ ^and IL-17 double-positive cells in mice receiving Com1-BMDCs were significantly higher than in mice receiving EnhA-pulsed BMDCs [41]. However, the percentages of regulatory T lymphocytes in mice receiving Com1-pulsed BMDCs were substantially lower than in animals receiving EnhA-pulsed BMDCs [41]. Our results suggest that the protection offered by Com1-pulsed BMDCs is correlated with the increased proliferation of Th1 CD4^+ ^T cells, preferential development of Th17 cells, and impaired expansion of regulatory T lymphocytes [41].

## Conclusions

Our results demonstrate that Com1 is able to induce complete maturation and activation of HMDCs that drive T cells toward Th1 and Tc1 polarization; however, HspB-induced incompletely mature HMDCs are unable to induce efficient T-cell proliferation and activation. Unlike HspB, Com1 is a potent protective antigen of *C. burnetii*, which has been demonstrated by adoptive transfers of Com1-pulsed and HspB-pulsed BMDCs into naive BALB/c mice.

## Methods

### Preparation of whole-cell antigen and recombinant proteins

*C. burnetii *Xinqiao strain (phase I) isolated from ticks in China [42] was propagated in embryonated eggs, inactivated with formalin, and purified by renografin density centrifugation, as described previously [16]. The purified coxiella cells were suspended in phosphate-buffered saline (PBS) buffer as a phase I antigen (I Ag). Recombinant proteins, Com1 and HspB, were prepared in our laboratory according to described methods [20]. Briefly, the *com1 *gene was amplified from *C. burnetii *(Xinqiao strain) genomic DNA with a pair of primers: primer 1 (5'-ATCGGATCCTTAGCCGGAACCTTGACC-3') with the *Bam*HI restriction site; and primer 2 (5'-GCCCTCGAGTAACGCTTTATTACCAATGACG-3') with the *Xho*I restriction site. The amplified gene fragment was cloned into a prokaryotic expression plasmid, pET-32a, by the restriction sites. *E. coli *BL21 cells were transformed by *com1*-recombined pET-32a. The *hspB *gene was amplified from *C. burnetii *genomic DNA with the other primer pair: primer 1 (5'-GCCGAGCTCGTGACGTTGGGACCAAAAGG-3') with the *Sac*I restriction site; and primer 2 (5'-ATACTGCAGCCGCCCATTCCTCCCATGCC-3') with the *Pst*I restriction site. The amplified gene fragment was cloned into a prokaryotic-expression plasmid pQE30 by the restriction sites.

*E. coli *M15 cells were transformed by *hspB*-recombined pQE30. The transformed bacterial cells were grown to an optical density (OD_600 _= 0.6) at 37°C in Luria-Bertani broth, containing 50 μg/ml ampicillin and then induced for 4 h with 0.5 mM isopropyl β-D-thiogalactoside (IPTG, Sigma-Aldrich, St Louis, MO, USA). The recombinant proteins containing six consecutive histidine residues were purified from the cellular debris of the transformed *E. coli *cells using the nickel-nitrilotriacetic resin affinity chromatography kit (QIAexpressionist, Qiagen GmbH, Germany) according to the manufacturer's instructions. The recombinant proteins Com1 and HspB were identified in SDS-PAGE (Figure 1A) and recognized using the sera from BALB/c mice experimentally infected with *C. burnetii *in an immunoblot assay (Figure 1B).

The purified recombinant proteins were treated with Triton X-114 (Amresco, Solon, OH, USA) to remove LPS, as described previously [43]. In brief, 5 μl Triton X-114 was added to 500 μl recombinant protein solution (1 μg/ml), and then the mixture was vigorously shaken in a vortex mixer for several seconds. The mixture was kept on ice for 5 min and then incubated at 37°C for 5 min. Finally, the mixture was briefly centrifuged, and the supernatant was collected. This procedure was repeated three times. Each LPS-removed protein was concentrated with a concentrator (VivaScience, Hannover, Germany), and the concentrated protein was diluted with the elution buffer in the affinity chromatography kit to make a protein solution (400 μg/ml). The recombinant proteins were used only when the LPS level was below 0.005 endotoxin units (EU)/ml, determined using the *Limulus *amoebocyte assay (Sigma-Aldrich, St Louis, MO, USA).

### Isolation and culture of cells

The human blood samples in this study were obtained from three healthy donors in our research group who clearly knew the purpose of this study, according to the Blood Donation Law of the People's Republic of China. This study was approved by the ethics committee of the Beijing Institute of Microbiology and Epidemiology.

HMDCs were generated from human peripheral blood mononuclear cells (PBMCs), as described previously [44]. In brief, PBMCs were isolated from blood buffy coats of healthy donors by Ficoll-Paque Plus (Haoyang Biotech, Beijing, China) density-gradient centrifugation, and the monocyte and T-cell fractions were obtained from PBMCs by Percoll density-gradient centrifugation (Haoyang Biotech, Beijing, China), according to the manufacturer's protocols. Residual lymphocytes were removed from the monocyte fraction by plastic adherence in 12-well plates (Costar, Corning, NY, USA), and the purity of the collected monocytes (CD14^+^) was greater than 90%, as determined by flow cytometry. The isogeneic T cells were collected from the supernatants, and more than 90% of the collected cells were CD3-positive in flow cytometric analysis.

The adherence monocytes were cultured with RPMI 1640 medium (Hyclone, Beijing, China) supplemented with 10% fetal bovine serum (FBS; Hyclone, Beijing, China), 50 ng/ml human IL-4 (PeproTech, Rocky Hill, NJ, USA), and 50 ng/ml human granulocyte-macrophage colony-stimulating factor (GM-CSF; PeproTech) at 37°C and 5% CO_2_. IL-4 and GM-CSF were added to the cultured cells every other day. After 5 days of culture, more than 95% of the cells had converted to immature HMDCs (iHMDCs), with a phenotype of CD11c^+^, CD54 ^low^, CD83 ^low^, CD40 ^low^, CD58 ^low^, CD86^low^, and CD80^low^, as determined by flow cytometry.

Approximately 1 × 10^6 ^iHMDCs was added to each well of a six-well plate; they were then treated with Com1 (20 μg/ml), HspB (20 μg/ml), *E. coli *LPS (6 μg/ml; Sigma-Aldrich, St Louis, MO, USA), and 50 μl elution buffer (mock pulse) for 24 h at 37°C. After 24 h of antigen stimulation, the HMDCs were harvested by centrifugation at 500 × g for 5 min, and their maturation and activation status were determined by flow cytometry, as described below.

### Flow cytometric assay

The harvested HMDCs were suspended in PBS containing 5% FBS, and the following monoclonal antibodies (mAbs) were used to characterize the cell phenotypes (BD Pharmingen, San Jose, CA, USA): anti-CD11c-APC, anti-CD58-FITC, anti-CD80-PE, anti-CD40-FITC, anti-CD83-PE, anti-CD54-PE, and anti-CD86-PE. In parallel, HMDCs were immunostained with the isotype-matched control mAb. Approximately 1 × 10^6 ^HMDCs were immunostained with three-color antibodies in 50 μl PBS containing 5% FBS. The HMDCs were incubated with different antibodies at room temperature for 15 min. After washing, 1% paraformaldehyde was added to fix the stained HMDCs. Samples were run on a FACSCalibur flow cytometer (BD Biosciences, San Jose, CA, USA) and analyzed using CellQuest software (BD Biosciences, San Jose, CA, USA).

### Intracellular cytokine detection by flow cytometry

After 20 h of antigen stimulation, HMDCs were incubated for 4 h with brefeldin A solution (1 μl/10^6 ^cells), and then the HMDCs were harvested and washed twice in PBS containing 1% bovine serum albumin (BSA). The brefeldin-treated HMDCs were stained with anti-CD11c-APC for 15 min at room temperature. After one fixing step with FACS lysing solution, HMDCs were permeabilized and immunostained with PE-conjugated anti-cytokine antibody (anti-IFN-α, anti-IL-12p70, or anti-IL-10) or isotype-matched control mAb for 30 min at room temperature.

After 20 h of incubation with the antigen-pulsed HMDCs, the T cells were incubated with brefeldin A for 4 h. After washing, the T cells were stained with anti-CD3-PerCP, anti-CD4-APC/anti-CD8-APC, anti-IFN-γ-FITC, anti-TNF-α-PE, or anti-IL-10-PE by the method described above. After washing, the stained cells were collected and suspended in PBS, and the different cytokine expression in HMDCs, CD4^+^, or CD8^+ ^cells was determined by flow cytometry.

### Measurement of early T-cell activation

After 12 h of incubation with the antigen-treated HMDCs, the T cells were stained with anti-CD3-PerCP, anti-CD4-APC/anti-CD8-APC, and anti-CD69-PE in 50 μl PBS containing 5% FBS at room temperature for 15 min. After washing, 1% paraformaldehyde was added to fix the stained cells, and then the CD69 expression on the CD4^+ ^or CD8^+ ^cells was determined by flow cytometry. In parallel, the cells were stained with isotype-matched control mAb.

### T-cell proliferation assay

Approximately 5 × 10^7 ^T cells in 1 ml PBS was mixed with 10 μM CFSE (Invitrogen, Eugene, OR, USA), and then the cells were incubated at 37°C for 10 min in the dark. After incubation, 10 ml PBS containing 5% FBS was added to each sample, followed by 5 min of incubation on ice. After three washes in PBS, the cells were harvested by centrifugation at 400 × g for 5 min. Approximately 1 × 10^5 ^CFSE-labelled responder cells was incubated with 1 × 10^4 ^antigen-pulsed HMDCs at 37°C and 5% CO_2 _for 96 h. The CFSE-labelled responder cells were stimulated with PHA (10 μg/ml) for 96 h as a positive control. After incubation, the cells were harvested and stained with anti-CD3-PerCP and anti-CD4-APC/anti-CD8-APC. CFSE-labelled responder cell proliferation in CD4^+ ^or CD8^+ ^cells was determined by flow cytometry, and the proliferation percentages of the T-cell fractions were calculated by ModFit software (BD Biosciences, San Jose, CA, USA).

### Immunoprotection analysis of Com1- and HspB-pulsed BMDCs

Mouse bone marrow dendritic cells (BMDCs, CD11c^+^) were isolated from the bone marrow of BALB/c mice, according to the protocol described previously [45]. Briefly, a single-cell suspension from the bone marrow was prepared from mouse femurs and cultured in a complete 1640 medium, containing 10% FBS, 100 μg/ml streptomycin sulfate, and 100 U/ml penicillin at 37°C and 5% CO_2_. Approximately 1 × 10^6 ^cells was added to each well of a six-well plate, and mouse GM-CSF (20 ng/ml; Peprotech) and IL-4 (10 ng/ml; Peprotech) was added to the culture medium every other day. After 6 days of culture, BMDCs were stimulated with *C. burnetii *I Ag (10 μg/ml), Com1 (10 μg/ml), HspB (10 μg/ml), *E. coli *LPS (2 μg/ml), or 25 μl elution buffer (mock pulse) for 24 h at 37°C and 5% CO_2_.

After antigen stimulation, the antigen-pulsed BMDCs were intraperitoneally (i.p.) injected into BALB/c mice (6 weeks age, female; 5 × 10^5 ^cells/mouse). Fourteen days after the BMDCs injection, mice were i.p. challenged with *C. burnetii *Xinqiao strain (1 × 10^6 ^cells/mouse) in Biosafety Level 3 Laboratory. Mice were sacrificed on day 7 post-challenge, and their spleens were harvested for detection of *C. burnetii *burden by a quantitative polymerase chain reaction (PCR) analysis [41,46]. For each mouse, 10 mg of spleen tissue was used to extract DNA with DNeasy Blood &Tissue kit (Qiagen, Germany), and the purified DNA was eluted from the column with 200 μl elution buffer. The data analysis was performed using SPSS software (version 10.0; SPSS). Group comparison was performed with the one-way analysis of variance (ANOVA) test. Values were expressed as means with standard deviations (SDs). *P *values of <0.05 were considered significant.

## Authors' contributions

YW carried out the analyses of HMDCs pulsed with antigens and T cells interacted with antigen-pulsed HMDCs and drafted the manuscript. XX performed the immune protective analysis of antigen-pulsed BMDCs against *C. burnetii *and drafted the manuscript. DW assisted the analyses of HMDCs and T-cells, and LW assisted the immune protective analysis of antigen-pulsed BMDCs. All authors read and approved the final manuscript.
